# Time course analysis based on gene expression profile and identification of target molecules for colorectal cancer

**DOI:** 10.1186/s12935-016-0296-3

**Published:** 2016-03-24

**Authors:** Guoting Chen, Ning Han, Guofeng Li, Xin Li, Guang Li, Zengchun Li, Qinchuan Li

**Affiliations:** Department of Emergency Surgery, East Hospital, Tongji University School of Medicine, No. 150, Jimo Road, Shanghai, 200120 China

**Keywords:** Cluster, Colorectal cancer, MicroRNA, Target, Transcription factor

## Abstract

**Background:**

The study aimed to investigate the expression changes of genes in colorectal cancer (CRC) and screen the potential molecular targets.

**Methods:**

The GSE37178 of mRNA expression profile including the CRC samples extracted by surgical resection and the paired normal samples was downloaded from Gene Expression Omnibus database. The genes whose expressions were changed at four different time points were screened and clustered using Mfuzz package. Then DAVID was used to perform the functional and pathway enrichment analysis for genes in different clusters. The protein–protein interaction (PPI) networks were constructed for genes in the clusters according to the STRING database. Furthermore, the related-transcription factors (TFs) and microRNAs (miRNAs) were obtained based on the resources in databases and then were combined with the PPI networks in each cluster to construct the integrated network containing genes, TFs and miRNAs.

**Results:**

As a result, 314 genes were clustered into four groups. Genes in cluster 1 and cluster 2 showed a decreasing trend, while genes in cluster 3 and cluster 4 presented an increasing trend. Then 18 TFs (e.g., TCF4, MEF2C and FOS) and 18 miRNAs (e.g., miR-382, miR-217, miR-1184, miR-326 and miR-330-5p) were identified and three integrated networks for cluster 1, 3, and 4 were constructed.

**Conclusions:**

The results implied that expression of *PITX2, VSNL1*, *TCF4*, *MEF2C* and *FOS* are time-related and associated with CRC development, accompanied by several miRNAs including miR-382, miR-217, miR-21, miR-1184, miR-326 and miR-330-5p. All of them might be used as potential diagnostic or therapeutic target molecules for CRC.

## Background

Colorectal cancer (CRC) is the third most common cancer worldwide with over 1,000,000 new cases every year [1]. In China, CRC is ranked as the fourth leading cause of cancer death with a pronounced increasing incidence during recent years [2]. Reportedly, the main risk factors for CRC include dietary and lifestyle factors, such as diet, obesity, physical activity, smoking and alcohol abuse [3–5]. Moreover, accumulating evidence indicate that multiple molecules are involved in CRC [6, 7]. For instance, *CARM1* has been linked to human CRC by modulating Wnt/β-catenin transcription and neoplastic transformation [8]. Several microRNAs (miRNAs) are also involved in the occurrence and development of CRC, such as miR-31, miR-126, miR-552, miR-592 and miR-224 [9, 10]. Moreover, p53-dependent expression of miR-34a is reported to suppress CRC progression by inhibiting an IL-6R/STAT3/miR-34a feedback loop [11]. More recently, utilizing the expression profile dataset of GSE44861, Wang et al. investigated the regulatory relationships between miRNAs and the target genes in CRC, and consequently identified a handful of crucial miRNAs such as miR-29 (with the putative target of *COL11A1*), miR-101 and miR-26 (both with the predicted targets of *PTGS2* and *ASPN*) [12]. However, pathogenesis of the cancer remains obscure.

Currently, time course gene expression is an increasingly popular approach for researching a wide range of biological processes (BPs) [13]. Recently, Musella et al. [14] have analyzed the gene expression profiling in human normal and CRC tissues at four time points after routine surgical procedure. They have identified several time-dependent genes in tumor and normal samples, such as *JUN*, *FOSB* and *ABL1*. However, they put emphasis on determining the critical time point for tissue handling in colon before which the gene alterations are not detected. Gene alteration trends during the whole period are not investigated. Besides, the potential interactions of the genes and the regulatory relationships between these genes and the related transcription factors (TFs) or miRNAs are not explored.

Therefore, we further investigated the mRNA expression profile of patients with CRC at four time points by re-analyzing the data of Musella et al. [14] which were deposited in Gene Expression Omnibus (GEO) database. The genes whose expressions were changed during different periods were screened and clustered. Then the gene ontology (GO) and kyoto encyclopedia of genes and genomes (KEGG) enrichment analyses were performed for genes in different clusters. Furthermore, the protein–protein interaction (PPI) networks were constructed and the TFs and miRNAs were screened based on the information in the relevant databases. Moreover, an integrated PPI-TF-miRNA regulatory network was established in each cluster. Our study sought to explore the changes of the critical time-related genes in CRC with potential regulators such as miRNAs and TFs, and provide evidence for molecular targets therapy of CRC.

## Methods

### Microarray data

The mRNA expression profile of GSE37178 was downloaded from GEO database (http://www.ncbi.nlm.nih.gov/geo/) deposited by Musella et al. [14]. The paired tumor and normal specimens collecting from 14 patients who underwent surgical resection at the INT-MI (Fondazione IRCCS Istituto Nazionale dei Tumori) were utilized in our study. The tumor specimens were all classified as moderately differentiated colonic Adenocarcinomas NOS, namely grade G2 basing on the American Joint Committee on Cancer 2010 (http://www.cancerstaging.org/) by the histological routinely examination [14]. The RNAs were extracted from the above patients. Six fragments from each patient were acquired and were randomly left at room temperature at four time points as follows: three fragments at <20 min (T_0_), one fragment at 60 min (T_1_), one fragment at 180 min (T_2_) and one fragment at 360 min (T_3_). Time was measured starting from patient’s surgical excision and the first time point (T_0_) was processed and frozen within 20 min from surgery. The control samples were the matched normal tissue at four time points after surgery [14]. In Musella’s study, all patients signed an informed consent and the experiments were approved by the Independent Ethical Committee of the INT-MI.

### Preprocessing of the raw data

Raw data were collected with Illumina Human HT-12 V3.0 expression beadchip (Illumina Inc, San Diego, CA, USA) and were preprocessed via background correction, quantile normalization and probe summarization using the LIMMA (Linear Models for Microarray Data, http://www.bioconductor.org/packages/release/bioc/html/limma.html) package [15] in R. Then the probe-level values in CEL files were converted into the mRNA expression values.

### Clustering analysis

The noise-robust soft clustering was performed for the samples in four time groups using Mfuzz package (http://www.bioconductor.org/packages/release/bioc/html/Mfuzz.html, version 2.6.1) [16]. According to the changes of gene expression at four different time points, genes were clustered into different groups. The default parameters were as follows: minimum standard deviation = 0.3, acore = 0.6.

### GO and KEGG pathway enrichment analysis

The DAVID (The Database for Annotation, Visualization and Integrated Discovery, http://david.abcc.Ncifcrf.gov/) online tool [17] was used to identify the over-represented GO categories in different BPs and the significant KEGG pathways for the genes in different clusters. The p value < 0.05 and gene count ≥ 2 were used as cut-off criteria for the selection of significant GO terms and KEGG pathways.

### Screening of transcription factors (TFs) and tumor-associated genes (TAGs)

The TFs were identified amongst the clustered gene sets according to the TRANSFAC database (http://www.gene-regulation.com/pub/databases.html), which collects eukaryotic TFs and their binding sites, as well as the DNA binding profiles [18]. Furthermore, oncogenes and tumor suppressor genes (TSGs) were also obtained from the gene sets by using the TSGene database (http://bioinfo.mc.vanderbilt.edu/TSGene/) [19] and TAG database (http://www.binfo.ncku.edu.tw/TAG/) [20].

### Network construction

The STRING (Search Tool for the Retrieval of Interacting Genes/Proteins, http://string-db.org/) database [21] was used to analyze PPIs for genes with the cut-off criterion of combined score >0.4 and the PPI network was then visualized by cytoscape (http://cytoscape.org/) software [22]. The protein product of a gene serves as a node in the PPI network, and the degree denotes the interplayed protein numbers of the specific protein. A node with high degrees is deemed as a hub node. By analyzing the connectivity degrees of the nodes in PPI networks, the hub proteins were obtained. Meanwhile, according to the miRNA-related databases such as miRanda (http://www.microrna.org/microrna/home.do), MirTarget2 (http://mirdb.org/miRDB/), PicTar (http://pictar.org/), PITA, TargetScan (http://www.targetscan.org/) and miRecords (http://mirecords.biolead.org/), the miRNAs which might regulate the genes were obtained with the cut-off criterion of p value <0.05. Then the miRNA-mRNA regulatory networks were constructed. Furthermore, based on the information in the ENCODE (encyclopedia of DNA elements) database (http://genome.ucsc.edu/ENCODE/), [23] the TF-target regulatory network was constructed. Finally, an integrated network combining the PPI network, miRNA-mRNA regulatory network and TF-target regulatory network was established for genes in each cluster.

## Results

### Clustering analysis

**Fig. 1:**
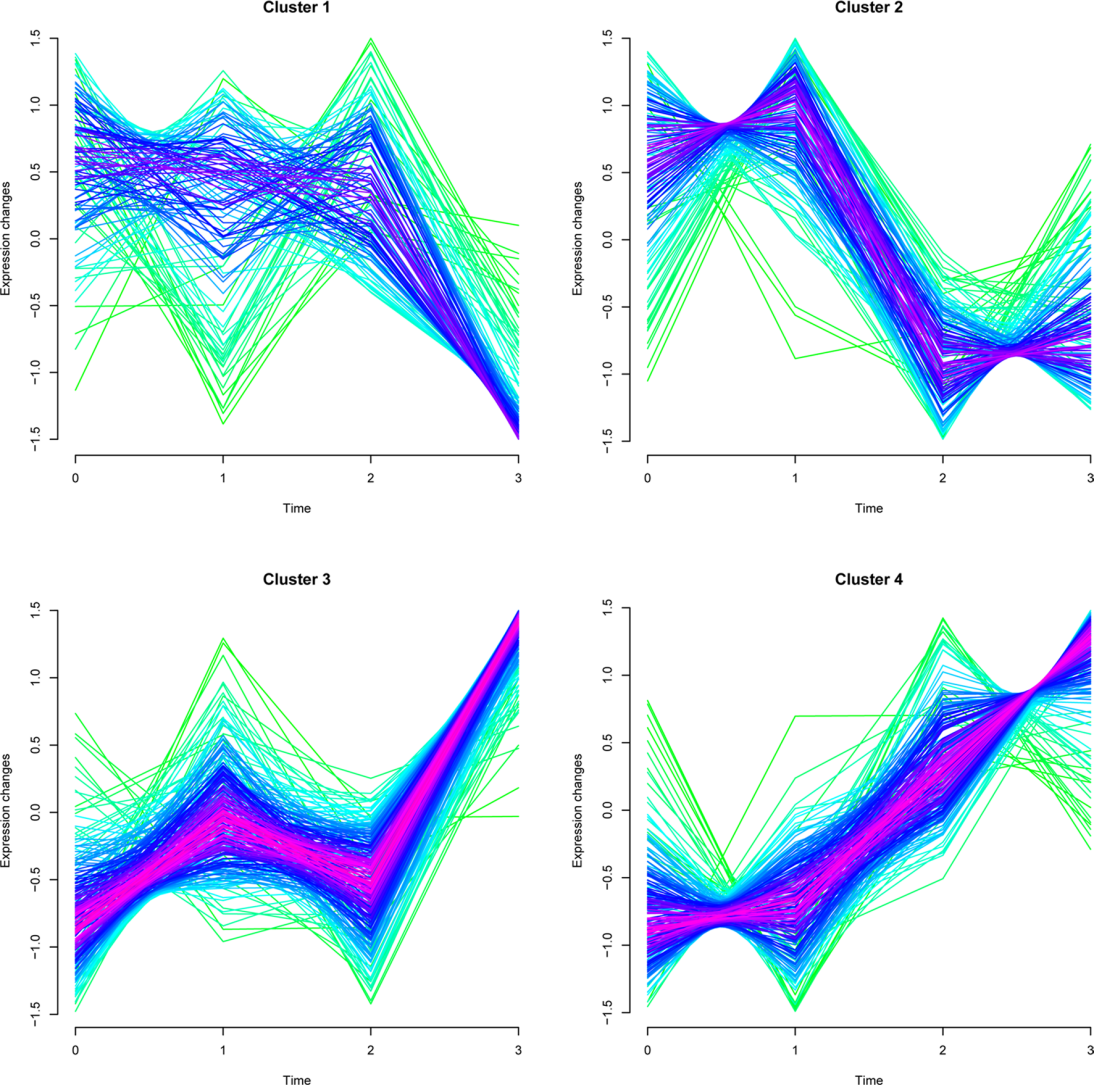
The gene expression changes in four clusters. The *color* varying from *green* to *red* represents that the trends of genes become more suitable to the changes of the cluster

With the aforementioned cut-off criteria, four gene sets were obtained. Among which, a downward expression trend with the increasing time for genes in cluster 1 (35 genes) and cluster 2 (53 genes) was observed, while an upward expression trend was presented in cluster 3 (121 genes) and cluster 4 (105 genes) (Fig. 1). In cluster 1, expressions of the genes were almost stable from T_0_ to T_2_, while were downward obviously from T_2_ to T_3_. In cluster 2, the expressions tended to be stable from T_0_ to T_1_, decreasing from T_1_ to T_2_, and then leveling out from T_2_ to T_3_. In contrast to cluster 1, expressions of genes in cluster 3 were stable from T_0_ to T_2_, while increasing from T_2_ to T_3_. In cluster 4, the expressions kept stable from T_0_ to T_1_ and were then increasing remarkably from T_1_ to T_3_.

### GO and KEGG enrichment analysis

The GO enrichment results showed that the genes in four clusters were enriched in 13, 5, 133 and 119 GO terms, respectively. The top ten GO terms in each cluster are listed in Table 1. Meanwhile, the enriched KEGG pathways of the genes in cluster 1, 3, and 4 were shown in Table 2. The results demostrated that metabolism of xenobiotics by cytochrome P450 (*P* = 9.06E−02), intestinal immune network for IgA production (*P* = 2.24E−08) and systemic lupus erythematosus (*P* = 5.81E−07) were the predominant KEGG pathways for genes in cluster 1, 3 and 4, respectively. Genes in cluster 2 were not enriched in any significant pathway.

**Table 1:** Enrichment analysis of top ten GO terms for genes in four clusters

	Term	Count	P value	Genes
Cluster 1	GO: 0006355~regulation of transcription, DNA-dependent	8	4.53E−02	*PITX2*, *MSX2*, *DLX3*, *IRX3*, *ZNF181*, *SMAD9*, *DMRT2*, *ETV4*
	GO: 0051252~regulation of RNA metabolic process	8	5.03E−02	*PITX2*, *MSX2*, *DLX3*, *IRX3*, *ZNF181*, *SMAD9*, *DMRT2*, *ETV4*
	GO: 0016337~cell–cell adhesion	3	9.79E−02	*REG3A*, *CLDN1*, *CDH3*
	GO: 0030574~collagen catabolic process	2	3.78E−02	*KLK6*, *MMP7*
	GO: 0044243~multicellular organismal catabolic process	2	4.88E−02	*KLK6*, *MMP7*
	GO: 0032963~collagen metabolic process	2	5.25E−02	*KLK6*, *MMP7*
	GO: 0044259~multicellular organismal macromolecule metabolic process	2	5.80E−02	*KLK6*, *MMP7*
	GO: 0044236~multicellular organismal metabolic process	2	6.88E−02	*KLK6*, *MMP7*
	GO: 0042552~myelination	2	7.06E−02	*KLK6*, *CLDN1*
	GO: 0007272~ensheathment of neurons	2	7.77E−02	*KLK6*, *CLDN1*
Cluster 2	GO: 0007586~digestion	3	2.69E−02	*CAPN9*, *TFF2*, *TFF1*
	GO: 0002526~acute inflammatory response	3	3.08E−02	*SERPINA1*, *C4BPA*, *LBP*
	GO: 0010038~response to metal ion	3	5.07E−02	*XDH*, *SERPINA1*, *TFF1*
	GO: 0045087~innate immune response	3	5.72E−02	*C4BPA*, *LBP*, *DEFB1*
	GO: 0008544~epidermis development	3	9.41E−02	*COL17A1*, *LAMA3*, *RBP2*
Cluster 3	GO: 0006955~immune response	30	3.03E−15	*ITGAL*, *IL16*, *LY86*
	GO: 0045321~leukocyte activation	16	1.67E−10	*ITGAL*, *CD3G*, *TNFSF4*
	GO: 0001775~cell activation	16	1.79E−09	*ITGAL*, *CD3G*, *TNFSF4*
	GO: 0006952~defense response	16	3.00E−05	*ITGAL*, *TNFSF4*, *AIF1*
	GO: 0042110~T cell activation	15	2.58E−13	*ITGAL*, *CD3G*, *TNFSF4*
	GO: 0046649~lymphocyte activation	15	1.35E−10	*ITGAL*, *CD3G*, *TNFSF4*
	GO: 0008283~cell proliferation	11	1.12E−03	*HHEX*, *STAT4*, *DOCK2*
	GO: 0042127~regulation of cell proliferation	11	5.52E−02	*HHEX*, *CORO1A*, *TNFSF4*
	GO: 0007610~behavior	10	6.45E−03	*CORO1A*, *DOCK2*, *IL16*
	GO: 0009611~response to wounding	10	1.37E−02	*ITGAL*, *TNFSF4*, *AIF1*
Cluster 4	GO: 0006334~nucleosome assembly	9	3.17E−08	*HIST2H2AA3*, *HIST4H4*, *HIST1H1E*
	GO: 0031497~chromatin assembly	9	4.19E−08	*HIST2H2AA3*, *HIST4H4*, *HIST1H1E*
	GO: 0065004~protein-DNA complex assembly	9	5.98E−08	*HIST2H2AA3*, *HIST4H4*, *HIST1H1E*
	GO: 0034728~nucleosome organization	9	7.10E−08	*HIST2H2AA3*, *HIST4H4*, *HIST1H1E*
	GO: 0006323~DNA packaging	9	4.24E−07	*HIST2H2AA3*, *HIST4H4*, *HIST1H1E*
	GO: 0007155~cell adhesion	18	5.34E−07	*EMCN*, *NRP1*, *CCL2*
	GO: 0022610~biological adhesion	18	5.45E−07	*EMCN*, *NRP1*, *CCL2*
	GO: 0006333~chromatin assembly or disassembly	9	7.96E−07	*HIST2H2AA3*, *HIST4H4*, *HIST1H1E*
	GO: 0009611~response to wounding	14	1.26E−05	*C3AR1*, *CCL3*, *IL6*
	GO: 0001568~blood vessel development	9	9.73E−05	*VEGFC*, *EMCN*, *NRP1*

*GO* gene ontology

**Table 2:** Enriched KEGG pathways for genes in four clusters

	Term	Count	P-value	Genes
Cluster 1	hsa00980: Metabolism of xenobiotics by cytochrome P450	2	9.06E−02	*CYP3A7*, *ALDH3B2*
	hsa00982: Drug metabolism	2	9.35E−02	*CYP3A7*, *ALDH3B2*
Cluster 3	hsa04672: Intestinal immune network for IgA production	9	2.24E−08	*TNFSF13B*, *CXCR4*, *ICOS*
	hsa04514: Cell adhesion molecules (CAMs)	9	4.80E−05	*ITGAL*, *SELL*, *ICOS*
	hsa04060: Cytokine-cytokine receptor interaction	9	4.66E−03	*IL2RB*, *TNFSF4*, *TNFSF13B*
	hsa05416: Viral myocarditis	8	6.22E−06	*ITGAL*, *MYH11*, *HLA*-*DPA1*
	hsa04062: Chemokine signaling pathway	7	1.14E−02	*DOCK2*, *CCL21*, *CXCR4*
	hsa05340: Primary immunodeficiency	6	2.37E−05	*CD3D*, *ICOS*, *TNFRSF13B*
	hsa04640: Hematopoietic cell lineage	6	1.71E−03	*CD37*, *CD3G*, *CD3D*
	hsa04660: T cell receptor signaling pathway	6	4.63E−03	*CD3G*, *CD3D*, *ICOS*
	hsa05330: Allograft rejection	5	4.50E−04	*HLA*-*DPA1*, *HLA*-*DPB1*, *HLA*-*DOA*
	hsa05332: Graft-versus-host disease	5	6.14E−04	*HLA*-*DPA1*, *HLA*-*DPB1*, *HLA*-*DOA*
	hsa04940: Type I diabetes mellitus	5	8.17E−04	*HLA*-*DPA1*, *HLA*-*DPB1*, *HLA*-*DOA*
	hsa05320: Autoimmune thyroid disease	5	1.70E−03	*HLA*-*DPA1*, *HLA*-*DPB1*, *HLA*-*DOA*
	hsa04612: Antigen processing and presentation	5	9.86E−03	*CD4*, *HLA*-*DPA1*, *HLA*-*DPB1*
	hsa05322: Systemic lupus erythematosus	5	1.79E−02	*HLA*-*DPA1*, *HLA*-*DPB1*, *HLA*-*DOA*
	hsa05310: Asthma	4	3.06E−03	*HLA*-*DPA1*, *HLA*-*DPB1*, *HLA*-*DOA*
Cluster 4	hsa05322: Systemic lupus erythematosus	9	5.81E−07	*HIST2H2AA3*, *HIST4H4*, *HIST1H2BC*
	hsa04670: Leukocyte transendothelial migration	6	1.89E−03	*HIST2H2AA3*, *HIST4H4*, *HIST1H2BC*
	hsa04514: Cell adhesion molecules (CAMs)	6	3.10E−03	*PECAM1*, *CLDN5*, *CLDN11*
	hsa04621: NOD-like receptor signaling pathway	4	1.15E−02	*IL6*, *CCL2*, *NFKBIA*, *NLRP3*
	hsa04620: Toll-like receptor signaling pathway	4	4.15E−02	*FOS*, *CCL3*, *IL6*, *NFKBIA*

*KEGG* Kyoto encyclopedia of genes and genomes

### Identification of oncogenes, TSGs and TFs amongst the selected genes

Basing on the relevant information in the aforementioned databases, potential oncogenes, TSGs and TFs amongst the selected genes in four clusters were revealed. As shown in Table 3, in cluster 1, *ETV4*, *MSX2*, *PITX2*, *SMAD9* were identified as TFs; *CUL7* was as a TSG and *KLK6* was an oncogene. In cluster 2, *CEACAM7*, *DEFB1*, *MUC1* and *SLC26A3* were considered as TSGs. In cluster 3, *EOMES*, *HEY2*, *HHEX*, *MEF2C*, *POU2AF1*, *STAT4*, *TCF21* and *TCF4* were TFs; *AIM2*, *BEX2*, *MAP4K1*, *PEG3*, *PRICKLE1*, *PYHIN1* and *TCF4* were TSGs. In cluster 4, *ATF3*, *DBP*, *HAND1*, *KLF2*, *MAFB* and *SOX18* were TFs and *FOS* was an oncogene.

**Table 3:** Transcription factors (TFs) and tumor associated genes (TAGs) for genes in four clusters

	TF	TSG	Oncogene	Other
Cluster 1	ETV4, MSX2, PITX2, SMAD9	CUL7	KLK6	
Cluster 2		CEACAM7, DEFB1, MUC1, SLC26A3		
Cluster 3	EOMES, HEY2, HHEX, MEF2C, POU2AF1, STAT4, TCF21, TCF4	AIM2, BEX2, MAP4K1, PEG3, PRICKLE1, PYHIN1, TCF4		EVI2B
Cluster 4	ATF3, DBP, HAND1, KLF2, MAFB, SOX18	ZFP36	FOS	CCL2, MAFB, RGS2, RHOB

*TSG* tumor suppress gene

### Screening of miRNAs

With the predefined selection criteria, several miRNAs which could target the genes were predicted. The results indicated that three miRNAs were predicted to target genes in cluster 1: hsa-miR-382 could target *CLDN1*, *DLX3* and *IRX3*; hsa-miR-217 could modeulate *DMRT2*, *MSX2*, *SMAD9* and *VSNL1*; hsa-miR-21 could regulate *PITX2* and *VSNL1*. In cluster 3, five miRNAs were obtained: *EOMES* and *RELN* were targeted by hsa-miR-935; *HET2* and *TCF4* were regulated by hsa-miR-1184; *C16orf45*, *HLA*-*DOA*, *PKIA*, *PTGIS*, *SPOCK1* and *TCF4* were targeted by hsa-miR-326. In cluster 4, ten miRNAs were obtained and the top three were hsa-miR-338-5p (the targets included *EBF3*, *KLF2* and *LDB2*), hsa-miR-656 (the targets included *COLEC12*, *DUSP1* and *TEK*) and hsa-miR-30d (the targets included *CYYR1*, *EBF3* and *EMCN*) (Table 4).

**Table 4:** The mircoRNAs (miRNAs) and their target genes

	MiRNA	Count	P-value	Gene
Cluster 1	hsa-miR-382	3	5.77E−03	*CLDN1*, *DLX3*, *IRX3*
	hsa-miR-217	4	1.11E−02	*DMRT2*, *MSX2*, *SMAD9*, *VSNL1*
	hsa-miR-21	2	4.80E−02	*PITX2*, *VSNL1*
Cluster 3	hsa-miR-935	2	1.69E−02	*EOMES*, *RELN*
	hsa-miR-1184	2	2.90E−02	*TCF4, HEY2*
	hsa-miR-326	6	3.02E−02	*TCF4, C16orf45*, *HLA*-*DOA*, *PKIA*, *PTGIS*, *SPOCK1*
	hsa-miR-496	5	3.45E−02	*RASGRP3*, *ARHGEF6*, *MOXD1*, *PEG3*, *PTGIS*
	hsa-miR-330-5p	5	4.22E−02	*TCF4, C16orf45*, *HLA*-*DOA*, *PKIA*, *SPOCK1*
Cluster 4	hsa-miR-338-5p	6	8.35E−04	*EBF3*, *KLF2*, *LDB2*, *NRP1*, *SLIT3*, *VEGFC*
	hsa-miR-656	4	6.96E−03	*COLEC12*, *DUSP1*, *TEK*, *ZFP36*
	hsa-miR-30d	9	1.08E−02	*CYYR1*, *EBF3*, *EMCN*, *GFPT2*, *NRP1*, *RGS2*, *RHOB*, *RRAD*, *ZNF521*
	hsa-miR-30b	9	1.10E−02	*CYYR1*, *EBF3*, *EMCN*, *GFPT2*, *NRP1*, *RGS2*, *RHOB*, *RRAD*, *ZNF521*
	hsa-miR-30a	9	1.11E−02	*CYYR1*, *EBF3*, *EMCN*, *GFPT2,NRP1*, *RGS2*, *RHOB*, *RRAD*, *ZNF521*
	hsa-miR-30c	9	1.12E−02	*CYYR1*, *EBF3*, *EMCN*, *GFPT2*, *NRP1*, *RGS2*, *RHOB*, *RRAD*, *ZNF521*
	hsa-miR-30e	9	1.12E −02	*CYYR1*, *EBF3*, *EMCN*, *GFPT2*, *NRP1*, *RGS2*, *RHOB*, *RRAD*, *ZNF521*
	hsa-miR-300	10	2.79E−02	*EBF3*, *IER5*, *LDB2*, *MMRN1*, *NFKBIA*, *NRP1*, *SELE*, *TEK*, *ZFP36*, *ZNF521*
	hsa-miR-27a	6	4.69E−02	*CDH5*, *EBF3*, *GEM*, *GFPT2*, *RGS1*, *VEGFC*
	hsa-miR-27b	6	4.73E−02	*CDH5*, *EBF3*, *GEM*, *GFPT2*, *RGS1*, *VEGFC*

### Analysis of integrated networks

**Fig. 2:**
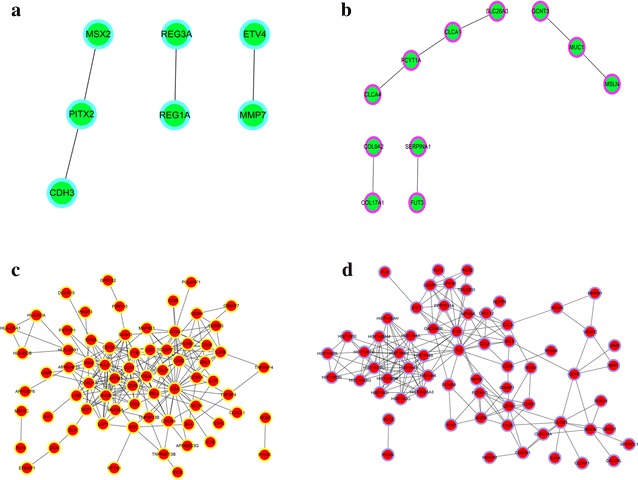
The protein–protein interaction (PPI) of genes in cluster 1 (**a**), cluster 2 (**b**), cluster 3 (**c**) and cluster 4 (**d**). *Green* and *red* nodes represent genes that were decreased and increased, respectively. The different *outer rings* indicate genes in different clusters

**Fig. 3:**
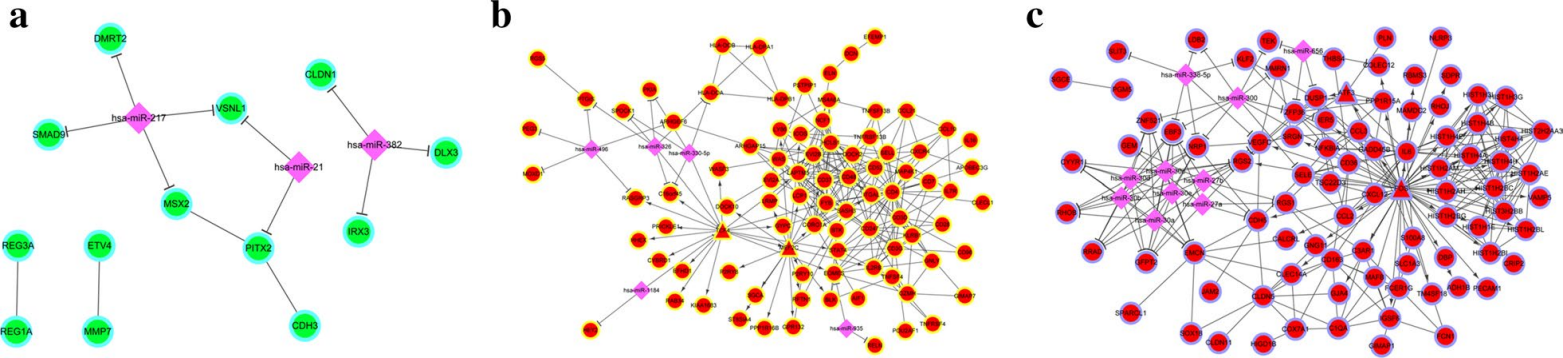
The integrated network of cluster 1 (**a**), cluster 3 (**b**) and cluster 4 (**c**). *Green* and *red* nodes represent genes that were decreased and increased, respectively. *Purple diamonds* represent miRNAs; *triangles* represent transcription factors

In order to explore the relationships among the genes, the PPI networks for genes in four clusters were constructed. As presented in Fig. 2, the network of cluster 1 contained 7 nodes and 4 PPIs; cluster 2 consisted of 11 nodes and 7 PPIs; cluster 3 contained 70 nodes and 219 PPIs; while cluster 4 comprised of 65 nodes and 187 PPIs. The hub nodes in four cluster were PITX2, PCYT1A, CD4 and FOS, respectively. Futhermore, the TF and miRNA regulatory networks were also constructed, respectively. The intergrated PPI-TF-miRNA networks were established for the genes in four clusters except in cluster 2, which did not contain the pronounced TFs and miRNAs. In the integrated network cluster (Int-c) 1, miR-217, miR-21, miR-382 and their respective target genes (e.g., *PITX2*, *VSNL1* and *DLX3*) were interacted with the PPI network in cluster 1 (Fig. 3a). In the Int-c3 network, five miRNAs (e.g., miR-935, miR-1184 and miR-326) and two TFs (MEF2C and TCF4) were integrated with the PPI network in cluster 3. The Int-c3 network showed that MEF2C could regulate 17 genes, such as *ITGAL*, *CORO1A* and *SGCA*; TCF4 could modulate 15 genes, such as *LAPTM5*, *FYB*, and *CYBRD1* (Fig. 3b). In the Int-c4 network, ten miRNAs (e.g., miR-338-5p, miR-656 and miR-30d) and two TFs (FOS and ATF3) were integrated with the PPI network in cluster 4. It was indicated that FOS could regulate 43 genes, such as *ATF3*, *IL6* and *HIST1H2BC*; ATF3 could modulate three genes, including *GADD45B*, *DUSP1* and *RGS2* (Fig. 3c).

## Discussion

In order to further investigate the pathogenesis and explore the molecular therapeutic methods for CRC, we re-analyzed the mRNA expression of CRC tissues extracted after surgical resection at four time points and screened a set of time-related genes, such as *PITX2* (paired-like homeodomain 2), *VSNL1* (visinin-like 1), *TCF4* (transcription factor 4), *MEF2C* (myocyte enhancer factor 2C) and *FOS* (FBJ murine osteosarcoma viral oncogene homolog). Meanwhile, *TCF4*, *MEF2C* and *FOS* were found to be TFs that have many target genes. Besides, a set of miRNAs such as miR-382, miR-217, miR-21, miR-1184, miR-326 and miR-330-5p were obatined.

In this study, the screened genes were classified into four clusters according to the gene expression change trends. Genes in cluster 1 and cluster 2 showed a decreasing trend during the period from T_2_ to T_3_, and from T_1_ to T_2_, respectively. In Int-c1 network, miR-382, miR-217 and miR-21 were shown to target several genes, such as *PITX2* and *VSNL1*.

*PITX2*, the hub node in PPI network 1, is shown to be involved in various functions such as tissue development by controlling cell growth in CRC [24]. Upregulation of *VSNL1* is an indicator of lymph node metastasis and poor prognosis in patients with CRC [25]. Another study also detects the elevated *PITX2* and *VSNL1* in CRC samples [26]. MiR-21, which is upregulated in many tumours, was predicted to target *PITX2* and *VSNL1* in our study. It has been found that plasma miR-21 is a potential diagnostic marker of CRC [27, 28]. The results collectively suggested that *PITX2* and *VSNL1* might be both the targets of miR-21 in CRC, and it might be the suppression of miR-21 that result in the downregulation of the two genes in our present study. In other cancers such as breast cancer and non small cell lung cancer, *PITX2* is also predicted as a target of miR-21, [29, 30] strengthening the reliability of our predictions and our faith to validate these targeting relationships in the follow-up studies using dual-luciferase reporter assay.

Besides, miR-382 was shown to target *CLDN1* (claudin 1), *DLX3* (distal-less homeobox 3) and *IRX3* (iroquois homeobox 3); miR-217 could regulate *VSNL1*, *MSX2* (msh homeobox 2), *DMRT2* (doublesex and mab-3 related transcription factor 2) and *SMAD9* (SMAD family member 9). Most of the targets were enriched in the GO terms of “regulation of transcription, DNA-dependent” and “regulation of RNA metabolic process”, indicating that the two BPs were mainly inhibited by miR-382 and miR-217.

Meanwhile, genes in cluster 3 and cluster 4 showed an increasing trend during the period from T_2_ to T_3_, and from T_1_ to T_3_, respectively. In cluster3, TCF4 was identified as an important TF, which regulated 15 genes, such as *C16orf45* (chromosome 16 open reading frame 45) and *RASGRP3* (RAS guanyl releasing protein 3 (calcium and DAG-regulated)). *C16orf45* was also targeted by miR-326 and miR-330-5p, and *RASGRP3* was regulated by miR-496. Besides, *TCF4* was also the target gene of miR-1184, miR-326 and miR-330-5p. It is found that the β-catenin/TCF4 complex, through its control over c-MYC and p21 activity, inhibits the differentiation on CRC cells [31]. Li et al. [32] found that miR-1184 was down-regulated and might play an important role in rectal cancer. Chen et al [33] reported that miR-326 was abnormally expressed in CRC and could be used as a novel biomarker for diagnosis of the cancer. Lin et al [34] showed that miR-330-5p was dysregulated in CRC with liver metastasis. In addition, TCF4 also regulates another TF in Int-c3 network, MEF2C, which could modulate 17 genes in cluster 3. MEF2C is a TF in the MEF2 family and involved in cardiac morphogenesis, myogenesis and vascular development. MEF2 has been shown to have a significant role in angiogenesis [35], and proven to be over-expressed in hepatocellular carcinoma [36]. Although there was little research mentioned that *C16orf45* and *RASGRP3* were directly associated with CRC, our results suggested miR-1184, miR-326 and miR-330-5p were involve in CRC by interacting with *TCF4*, *C16orf45* and *RASGRP3*. Therefore, it might be inferred that *TCF4*, *C16orf45* and *RASGRP3*, mediated by these miRNAs, might play important roles in the CRC progression and be used as the therapeutic targets. The predicted regulation of *TCF4* and three miRNAs, miR-1184, miR-326 and miR-330-5p, will be validated in our further studies.

FOS, the hub node in the PPI network 4, is proved to be a TF and regulate 43 genes in cluster 4. FOS is found to interact with JUN (jun proto-oncogene, c-JUN) to form the transcription factor AP-1 (activating protein 1) that is crucial for cell adaptation to environmental changes [37, 38]. Meanwhile, Musella et al. [14] also have found that *JUN* is a critical time-related gene in CRC. AP-1 regulates the expression of multiple genes essential for cell differentiation, proliferation, and apoptosis and plays an important role in various human diseases such as CRC [39]. Therefore, our results suggested that the increase of FOS may play important role in the period of CRC patients after surgery.

However, there are some limitations in our study. First, we mainly analyzed the relationships among genes and miRNAs in the same cluster. The regulatory relationships among molecules in different clusters and the interactions between clusters are needed further explored. Second, substantial experimental validations of the predicted miRNA-target relationships are warranted to confirm our results. Nevertheless, our results are still of great value to provide clues for further studies focusing on roles of several special miRNAs, such as miR-21, -1184, -330-5p and -326 in CRC progression.

## Conclusions

In summary, a set of genes and the related TFs and miRNAs were screened by analyzing of time course gene expression in CRC tissues. Our findings suggested that expression of genes such as *PITX2, VSNL1*, *TCF4*, *MEF2C* and *FOS*, are time-related. Meanwhile, miRNAs such as miR-382, miR-217, miR-21, miR-1184, miR-326 and miR-330-5p might be involved in the progression of CRC. All the genes and miRNAs might be used as potential diagnostic and/or therapeutic target molecules for CRC.
